# Supplementary material for: CRISPR/Cas9-Mediated SlATG5 Mutagenesis Reduces the Resistance of Tomato Fruit to Botrytis cinerea
Source: Foods. 2023 Jul 19;12(14):2750. doi: 10.3390/foods12142750 (PMC10380010; doi:10.3390/foods12142750)
Supplement: Supplementary file 1 [file foods-12-02750-s001.zip › foods-2477271-supplementary.pdf]

**Table S1. Primers used in recombinant pYLCRISPR/Cas9 vector construction**

| Purpose | Primers | Sequence (5'→3') |
|---|---|---|
| 1st PCR | U-F | CTCCGTTTTACCTGTGGAATCG |
| | gR-R | CGGAGGAAAATTCCATCCAC |
| | A5gRT1+ | GAGGCAGGTACCCAATTCGGTTTTAGAGCTAGAAAT |
| | A5AtU3bT1- | CGAATTGGGTACCTGCCTCTGACCAATGTTGCTCC |
| | A5gRT2+ | TGGAGCGCCAGAGTTCTAATGTTTTAGAGCTAGAAAT |
| | A5AtU3dT2- | ATTAGAACTCTGGCGCTCCATGACCAATGGTGCTTTG |
| 2nd PCR | Pps-GGL | TTCAGAGGTCTCTCTCGACTAGTATGGAATCGGCAGCAAAGG |
| | Pgs-GG2 | AGCGTGGGTCTCGTCAGGGTCCATCCACTCCAAGCTC |
| | Pps-GG2 | TTCAGAGGTCTCTCTGACACTGGAATCGGCAGCAAAGG |
| | Pgs-GGR | AGCGTGGGTCTCGACCGACGCGTATCCATCCACTCCAAGCTC |

The restriction enzyme *Bsa*I site was underlined.

**Table S2. Oligonucleotide primers used in mutation detection**

| Purpose | Primer | Sequence (5'→3') |
|---|---|---|
| Transgenic plant identification | Hyg for | CTTGACATTGGGGAGTTTAGCGAGA |
| | Hyg rev | CCCTTATCTGGGAACTACTCACACA |
| Mutation detection | ATG5-T1-F | CTCTCCACCAGACTCTCTCT |
| | ATG5-T1-R | TCCAGTACAATCCACGTACT |
| | T1 seq | CACACACACACATATATATAGAGAG |
| | ATG5-T2-F | CTGTGGGACTATGTAGGTTG |
| | ATG5-T2-R | TTTTGTGTTCTGATGGTGTT |
| | T2 seq | AGATGTGCGATTGGGTTTTACAATC |

**Table S3. Sequences of Specific Primers Used for qPCR Analysis**

| name | accession no. | forward primer (5′ → 3′) | reverse primer (5′ → 3′) |
|---|---|---|---|
| *β*-actin | NM_001308447 | CAGCAGATGTGGATCTCAAA | CTGTGGACAATGGAAGGAC |
| *SlEDS1* | NM_001320249 | GGAATTGAAGTCAGAGATGAGCTAA | AAAGTTCCAGCAAAAGCAAAAA |
| *SlPAD4* | XM_019212160 | CCGTGATCAGATGGTAGAAATAATG | CGGCAGAGAAGCCAGAGAGT |
| *SlPR1* | NM_001247429 | TGGTATTAGCCATATTTCAC | CCAGTTGCCTACAGGATC |
| *SlJAZ1* | NM_001247954 | CGAGACGGAATTCACTTACAAGA | TGAGCACCTAATCCCAACCAT |
| *SlMYC2* | NM_001324483 | GGAGGCGAAGACTCTGAACATT | GCTGGCTTTCTACCTCGCTTC |
| *SlLOXD* | NM_001320292 | CGAACTTGAAAACAGAGCGA | GTAATACTCTCCAGAAAGAACTCCT |
| *SlNPR1* | NM_001247629 | TGTTTTATGTGGATTGGTGGCT | CTTCTGCTTGATGGGATGACTG |
| *SlATG5* | XM_010317407 | AAGAGCAACACGGAACGAAGT | TACCACCCATGCAAAAGGAAT |

**Table S4. Editing type of *slatg5* mutants in T0 generation**

| | | |
|---|---|---|
| CR-ATG5-2<br>(Biallelic) | Target 1 | Reference: ATTGCAGATTTTAGCTCCTCGAATTGGGTACCTGCCTCTTTTAGCACAAAAAGTA<br>Allele1: ATTGCAGATTTTAGCTCCTCCAATTGGGTACCTGCCTCTTTTAGC (substitution)<br>Allele2: ATTGCAGATTTTAGCTCCTC (12-bp del) CTGCCTCTTTT (573-bp del) ATCAC (deletion) |
| CR-ATG5-3<br>(Biallelic) | Target 2 | Reference:ATATGTCCCAATCTGACCAATTAGAACTCTGGCGCTCCATTATGG<br>Allele1:ATATGTCCCAATCTGACCAA--AGAACTCTGGCGCTCCATTATGG(deletion)<br>Allele2:ATATGTCCCAATCTGACCAATTTCAGCCCCGCCAT (complicated variant) |
| CR-ATG5-7<br>(heterozygous) | Target 2 | Reference:TGAATATGTCCCAATCTGACCAATTAGAACTCTGGCGCTCCATTATGG<br>Allele1:TGAATATGTCCCAATCTGACCAATTAGAACTCTGGCGCTCCATTATGG (WT)<br>Allele2:TGAATATGTCCCAATCT--------------CTGGCGCTCCAT (deletion) |

Target sequences are shown in red letters. Deletion is denoted in green. Insertion is expressed with a green letter and substitution is shown in a blue letter. Complex variant regions marked yellow.

**Table S5. CRISPR/Cas9-Mediated Mutagenesis and Transmission from T0 to T1 Generation**

| Transgenic line | T0 | | T1 mutation segregation | | | | |
|---|---|---|---|---|---|---|---|
| | Zygosity | Genotype | No. of plants tested | Wt | Biallele | Homozygote | Heterozygote |
| L2 | T1 (Bia) | s1, d585 | 20 | 7 | 8 (s1, d585) | 0 | 3 (wt, s1)<br>2 (wt, d12) |
| L3 | T2 (Bia) | d1, variant | 8 | 8 | 0 | 0 | 0 |
| L7 | T2 (He) | wt, d14 | 10 | 3 | 3 (d14, s1) | 0 | 4 (wt, d14) |

d#, the number of bases deleted from the target sequences; s#, the number of bases substituted origin target sequences; wt, wild-type sequence without mutations detected at target sequences.
